# Upregulation of miR-3195, miR-3687 and miR-4417 is associated with castration-resistant prostate cancer

**DOI:** 10.1007/s00345-021-03723-4

**Published:** 2021-05-14

**Authors:** C. G. H. Rönnau, S. Fussek, F. P. Smit, T. W. Aalders, O. van Hooij, P. M. C. Pinto, M. Burchardt, J. A. Schalken, G. W. Verhaegh

**Affiliations:** 1 Urological Research Laboratory, Department of Urology, Radboud University Medical Center, PO Box 9101, 6500 HB Nijmegen, the Netherlands; 2 Department of Urology, University Medicine, Greifswald, Germany; 3 MDxHealth BV, Nijmegen, The Netherlands; 4 Radboud Institute for Molecular Life Sciences, Nijmegen, The Netherlands

**Keywords:** Prostate cancer, Castration-resistance, MicroRNA, Androgens

## Abstract

**Purpose:**

Prostate cancer (PCa) is a leading cause of cancer-related death. Upon androgen-deprivation therapy, the disease may progress further to castration-resistant PCa (CRPC) with a poor prognosis. MicroRNAs (miRNAs) are small non-coding RNAs, which play crucial roles in gene regulation. The aim of our study is to find CRPC-associated miRNAs and to evaluate their functional role.

**Methods:**

In this study, 23 benign prostatic hyperplasia (BPH), 76 primary PCa, and 35 CRPC specimens were included. Total RNA extracted from tissue sections was used for miRNA profiling on the Affymetrix GSC 3000 platform. Subsequently, stem-loop RT-qPCR analysis was performed to validate the expression levels of selected miRNAs. PCa cell lines were transfected with miRNA mimics or inhibitors to evaluate the effects on cell proliferation, cell migration and cell invasion.

**Results:**

In our profiling study, several miRNAs were found to be deregulated in CRPC compared to primary PCa tissue, of which miR-205 (− 4.5-fold; *p* = 0.0009), miR-92b (− 3.1 fold; *p* < 0.0001) were downregulated and miR-3195 (5.6-fold; *p* < 0.0001), miR-3687 (8.7-fold; *p* = 0.0006) and miR-4417 (5.0-fold; *p* = 0.0005) were most upregulated. While *KLK3*, miR-21 and miR-141 expression levels in androgen-treated VCaP and LNCaP cells were increased, the expression levels of miR-3687 and miR-4417 were reduced. None of the miRNAs were androgen-regulated in the AR-negative PC3 cell line. Overexpression of miR-3687 reduced cell migration and cell invasion, whilst miR-3195 enhanced cell migration.

**Conclusion:**

We have identified several novel deregulated miRNAs in CRPC tissue, including two microRNAs that are potentially involved in tumor invasion. Our data support the hypothesized involvement of miRNAs in PCa tumorigenesis and progression to CRPC. The applicability of these miRNAs as novel biomarkers for CRPC remains to be further investigated.

**Supplementary Information:**

The online version contains supplementary material available at 10.1007/s00345-021-03723-4.

## Introduction

The main treatment for metastatic prostate cancer (PCa) is androgen-deprivation therapy (ADT). Unfortunately, in most patients tumor cells become resistant to ADT and patients progress to so-called castration-resistant PCa (CRPC) and die within 2–4 years [1]. A better understanding of the pathogenesis of CRPC and new treatment modalities are urgently needed. So far, research has focused primarily on the deregulation of the androgen receptor (AR) signaling axis. These investigations culminated with the development of a number of new and more potent drugs to suppress AR signaling, such as abiraterone acetate or enzalutamide [2].

Non-coding RNAs (ncRNAs) play essential roles in key biological processes and can be involved in cancer pathogenesis [3, 4]. MicroRNAs (miRNAs) are small ncRNAs that regulate gene expression at the posttranscriptional level by binding to the 3′UTR of target mRNAs leading to inhibition of mRNA translation and to mRNA degradation [4, 5]. Many of the more than 2500 human miRNAs are aberrantly expressed in human cancer, and these miRNAs can act as oncomiRs or tumor suppressors [6, 7].

Our understanding of miRNA dysregulation in CRPC and their functional role in disease development and progression is beginning to take shape, but is still far from complete. Some miRNAs are regulated via the androgen receptor (AR), while others regulate AR expression [8, 9]. Seven androgen-regulated miRNAs, miR-21, miR-32, miR-99a, miR-99b, miR-148a, miR-221 and miR-590-5p, have been reported to be differentially expressed in CRPC compared to benign prostate hyperplasia (BPH) [10]. Furthermore, increased expression of miR-19a in CRPC tissue has been reported, and the latter miRNA regulates proliferation and apoptosis by direct targeting of the *BTG1* tumor suppressor gene [11]. Recently, overexpression of miR-1247-5p in CRPC tissue targeting MYC-binding protein 2 has been shown [12]. Unfortunately, there is little consensus in the identified CRPC-associated miRNAs.

The identification of CRPC-associated miRNAs, and especially those who promote disease progression, will lead to a better understanding of the molecular alterations associated with the pathogenesis and progression of CRPC. The purpose of the present study was to discover and validate CRPC-associated miRNAs and to evaluate their function in CRPC progression.

## Materials and methods

### Human tissue samples

The collection of human tissue samples (Table 1) was approved by the local ethical committee of the Radboud university medical center. BPH specimens were obtained by transurethral resection of the prostate (TURP), primary PCa tissue by radical prostatectomy and CRPC tissues by TURP after local progression under ADT (as defined by EAU guidelines). Tumors were classified as low grade (Gleason score ≤ 6) and high grade (Gleason score ≥ 7). All specimens were snap frozen in liquid nitrogen and stored until use. Hematoxylin- and eosin-stained tissue sections were used to determine the differentiation grade of tumor tissue and the percentage of epithelium and stroma.

**Table 1:** Demographic characteristics of the patients enrolled in this study

Profiling (microarray analysis)					
	All cases	BPH	LG	HG	CRPC
	(*n* = 51)	(*n* = 3)	(*n* = 16)	(*n* = 17)	(*n* = 15)
Age, median (IQR)	66.75 (53–82)	66.67 (61–72)	63.25 (55–73)	63.65 (53–75)	74.00 (62–82)
PSA in ng/ml, median (IQR)			17.57 (2.4–63)	17.37(1.1–74)	
Missing PSA			2	1	

Validation (SL-RT-qPCR)					
	All cases	BPH	LG	HG	CRPC
	(*n* = 83)	(*n* = 20)	(*n* = 20)	(*n* = 23)	(*n* = 20)
Age, median (IQR)	65.65 (53–83)	65.60 (55–79)	62.75 (54–69)	63.35 (53–69)	71.25 (59–83)
PSA in ng/ml, median (IQR)			13.62 (2.8–24.0)	20.58 (7.6–55)	
Missing PSA			5	8	

*BPH* benign prostate hyperplasia, *CRPC* castration-resistant prostate cancer, *HG* high grade prostate cancer, *IQR* interquartile range, *LG* low grade prostate cancer, *PSA* (serum) prostate-specific antigen

### Microarray analysis

Total RNA was extracted from 20-µm tissue sections using TRIzol reagent (Life Technologies). RNA quantity and quality were assessed on a NanoDrop 1000 and an Agilent 2100 Bioanalyzer. MiRNA profiling was performed on the Affymetrix GSC 3000 platform using GeneChip® miRNA 3.0 Arrays, according to the manufacturer’s instructions. Data were analyzed using the Genomics Suite software package (Partek). MiRNA expression levels of individual samples were calculated, and the results were sorted according to the fold change and significance (ANOVA test) between different groups.

### Real-time PCR analysis

MiRNA expression was determined by stem-loop RT-qPCR analysis using 100 ng total RNA [13]. Real-time PCR was performed using 2 µl (*i.e.* 10%) RT product, miRNA-specific primers (Suppl. Table 1) and SYBR Green PCR Mix (Roche). The qPCR was performed on a LightCycler LC480 Instrument (Roche), using default amplification conditions. Melting curves were analyzed and Cp-values were calculated using the LightCycler 480 Software. MiRNA expression levels were normalized to the weighted average of the expression of RNU6, miR-26a and miR-107.

For gene expression analysis, total 2 μg RNA was DNase-I-treated and cDNA was synthesized using random hexamer primers and SuperScript II Reverse Transcriptase (Life Technologies). Real-time PCR was performed essentially as described above. Gene expression levels were normalized to *HPRT1* housekeeping gene levels, and relative gene expression was calculated using the $\Delta \Delta C_{{\text{t}}}$ method.

### Cell culture and androgen stimulation

The prostate cancer-derived cell lines, LNCaP (ATCC# CRL-1740) and PC-3 (ATCC# CRL-1435), and the CRPC-derived cell lines, DuCaP and VCaP (provided by dr. Kenneth Pienta, Johns Hopkins, Baltimore, USA), were cultured in RPMI-1640 medium (Invitrogen), supplemented with 10% fetal bovine serum (Sigma, F7524). Cultures were maintained in a humidified atmosphere at 37 °C and 5% CO_2_. For androgen stimulation, cells were cultured for 3 days in medium supplemented with charcoal-stripped (i.e. androgen-free) serum (CSS), and then cells were treated with R1881 (MSD-Organon). Cell lines were authenticated using the PowerPlex 21 system (Promega) by Eurofins Genomics (Germany), and cells were frequently tested for *Mycoplasma* infection.

### Pre-miR miRNA mimic and anti-miR miRNA inhibitor transfection

Cell lines were transiently transfected with 20–50 nM of pre-miR miRNA precursors (‘mimics’) and anti-miR miRNA inhibitors (Ambion; Supplementary Table 2). For transfection, Lipofectamine RNAiMAX transfection reagent (Life Technologies) was used, according to the manufacturer’s instructions.

### Cell viability and apoptosis analysis

Cells were seeded 48 h after transfection in 96-well plates at a density of 10,000 cells per well (VCaP) or 3000 cells per well (PC3). At several time points, CellTiter-Glo (Promega) cell viability assays were performed, and luminescence was measured on a Victor 3 Multilabel Plate Reader (PerkinElmer). Apoptosis in transfected PC3 cells was measured using the Apo-ONE Caspase-3/7 Assay (Promega). Both assays were performed according to manufacturer's instructions.

### Cell migration assay

Cell migration was determined using scratch assays. PC3 cells were seeded in 6-well plates at a density of 600,000 cells per well and grown to confluence. Two days after transfection, a cell-free wound area was created by scratching the cells with a 1-mL pipette tip. Medium was replaced and cells were allowed to migrate into the cell-free area. Microscopic images were taken at regular intervals and used to measure the speed of cell migration.

### Cell invasion assay

Cell invasion was investigated using Matrigel pre-coated invasion chambers with 8-μm pore polycarbonate membranes (BD Biosciences). Transfected cells were transferred to the upper chamber in serum-free medium, and the lower compartment contained 10% FCS as chemo-attractant. Cells were allowed to invade the Matrigel and migrate through the pores for 24 h, and invading cells were quantified using CellTiter-Glo reagent (Promega).

### Data analysis

Statistical analyses were performed using Graphpad Prism 5. Receiver operator characteristic (ROC) analysis and area under the curve (AUC) calculation were performed using IBM SPSS v20. P-values were calculated using ANOVA or unpaired two-tailed *t*-test and expression profiling data were adjusted using Bonferroni correction for multiple testing. *p* values < 0.05 or < 0.0026 (after Bonferroni correction) were considered statistically significant. NormFinder software was used for the identification of optimal normalization miRNAs.

## Results

### Characteristics of patients enrolled in this study

Clinical characteristics of the patients enrolled in this study, including age and tumor grade and stage, are described in Supplementary Tables 3 and 4. We included 35 CRPC patients (*n* = 15 for microarray and *n* = 20 for qPCR analysis). In the discovery cohort, four patients got an orchiectomy and 11 patients received ADT, and in the validation cohort 10 patients received an orchiectomy and 10 patients received ADT.

### Discovery of deregulated microRNAs in CRPC tissue

GeneChip® miRNA 3.0 Array analysis was performed to detect and evaluate the expression of more than 1700 human mature miRNAs and more than 2000 snoRNAs and other small ncRNAs in prostate cancer tissues. The main focus was to identify differentially expressed miRNAs in CRPC compared to primary PCa (Suppl. Table 4). We identified 26 miRNAs that were ≥ 1.5 times upregulated and 125 miRNAs that were ≥ 1.5 times downregulated in CRPC (Supplementary Figure 1; Supplementary Table 5). In addition, we compared the expression levels of small ncRNAs in PCa and BPH specimens (Supplementary Table 5). Based on the fold-change and significance between the cancer and control groups, several miRNAs and snoRNAs were selected for further validation (Table 2).

**Table 2:** Differentially expression of small ncRNAs in CRPC vs primary prostate cancer tissue

CRPC vs. PCa		
ncRNA	Fold change	p-value
miR-3687	+ 8.72	0.0006
miR-3195	+ 5.56	< 0.0001
miR-4417	+ 5.04	0.0005
miR-451	+ 3.85	< 0.0001
SNORD78	+ 2.47	0.0143
miR-3156-5p	+ 2.17	0.0040
miR-194	+ 1.80	0.0057
SNORA84	+ 1.67	0.1064
sno-miR-78	+ 1.46	0.1120
miR-4521	+ 1.41	0.0663
miR-4286	+ 1.28	0.3183
miR-3651	+ 1.21	0.4377
SNORD49A	+ 1.20	0.3018
miR-3609	+ 1.15	0.5245
miR-708	+ 1.08	0.6185
miR-210	− 1.05	0.9146
miR-183	− 1.10	0.7044
miR-92b	-− 3.10	< 0.0001
miR-205	− 4.47	0.0009

*CRPC* castration-resistant prostate cancer, +  upregulated ncRNAs in CRPC compared to primary prostate cancer tissue (PCa), – downregulated ncRNAs in CRPC compared to primary prostate cancer

*p* values were calculated based on the unpaired two-tailed *t*-test, with Bonferroni correction

### Analysis of putative reference miRNAs in prostate cancer tissue

Although small RNAs such as the U6 small nuclear RNA (RNU6) are generally used for normalization, no *bona fide* reference miRNA for PCa tissue has been validated yet. Three miRNAs, miR-26a, miR-107 and miR-151-5p, were expressed at comparable levels in all investigated tissue specimens in the microarray analysis (Supplementary Fig. 2a, c and e). These miRNAs were validated in the same tissue samples (*n* = 5 for each group), and in an independent cohort containing 93 additional tissue samples, using stem-loop RT-qPCR. Also in qPCR analysis, the three miRNAs showed stable expression levels (Supplementary Figs. 2b, d, f, h and 3a–c). NormFinder analysis revealed that the stability values of the miRNAs were very similar and that the combination of miR-26a, miR-107 and RNU6 was statistically superior to the use of RNU6 alone (Supplementary Figs. 2g and 3d). Therefore, the weighted average miR-26a, miR-107, and RNU6 expression was used for normalization purposes.

### Validation of differentially expressed miRNAs in CRPC

**Fig. 1:**
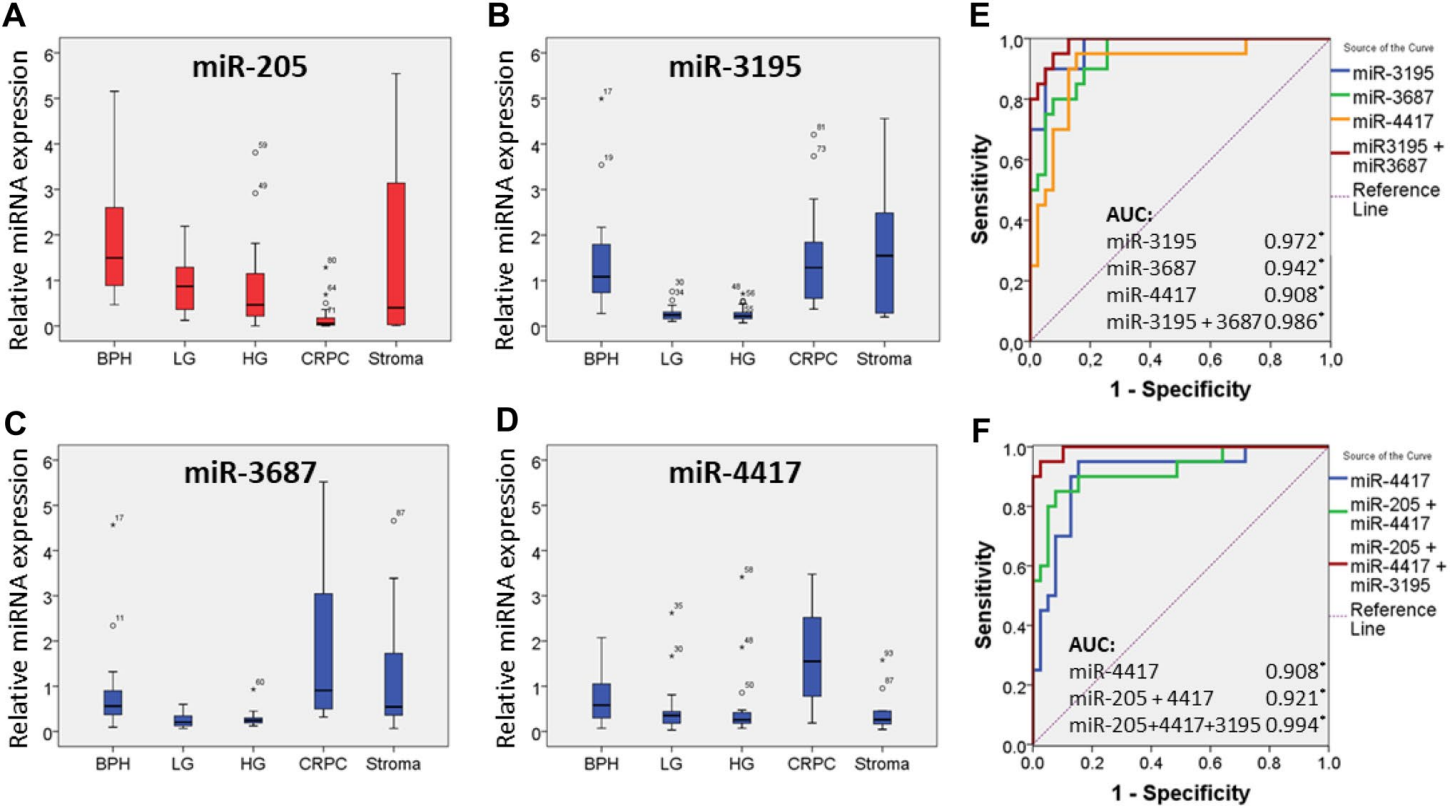
Expression of miR-205, miR-3195, miR-3687, and miR-4417 in human prostate tissue and their association with CRPC. MiRNA expression analysis was performed by stem-loop RT-qPCR analysis. The results were normalized to the weighted average of miR-26a, miR-107 and RNU6 levels. The median expression and the standard deviation are shown; outliers are indicated by open circles and asterisks. MiR-205 **a** is significantly downregulated, whilst miR-3195 **b**, miR-3687 **c** and miR-4417 **d** are significantly upregulated in CRPC tissue compared to primary PCa tissue. BPH, benign prostate hyperplasia; LG, low grade prostate cancer; HG, high grade prostate cancer; CRPC, castration-resistant prostate cancer. **e**, **f** Receiver operating characteristic curve (ROC) analysis was performed using miRNA expression data from the validation set (*n* = 83), comparing the ability of miR-3195, miR-3687, miR-4417 alone and combinations of miR-3195 and miR-3687, and of miR-205, miR-3195 and miR-4417 to identify men with CRPC. Areas under the curve (AUC) are shown (*p < 0.01)

Selected small ncRNAs were validated in a larger independent cohort of tissue samples using stem-loop RT-qPCR analysis. Tissue samples included BPH (*n* = 20), low and high grade PCa (*n* = 20 and *n* = 23, resp.), CRPC (*n* = 20) and stroma (*n* = 10). The stroma samples from BPH (*n* = 6) and cancer (*n* = 4) contained less than 1% of epithelial cells. We found a significant upregulation of miR-3195, miR-3687, miR-4417, miR-451, miR-3156-5p and miR-194 in CRPC compared to primary PCa (Table 2; Fig. 1, Supplementary Fig. 4). MiR-3195, miR-3687 and miR-4417 were the most upregulated CRPC-associated miRNAs (Table 2). MiRNA-205 was the most significantly downregulated miRNA in CRPC compared to primary PCa tissue (Fig. 1a; Supplementary Table 4), confirming earlier reported findings [14]. MiR-92b, a known oncogenic miRNA [15, 16], is significantly upregulated in primary PCa versus BPH tissue. Surprisingly, this miRNA is significantly downregulated in CRPC tissue compared to primary PCa (Table 2; Supplementary Fig. 4a). In addition to the miRNAs, also SNORD78 was significantly deregulated (2.47-fold up, *p* < 0.01) in CRPC (Table 2, Supplementary Fig. 4i).

### ROC curve analysis

The ability of miR-3195, miR-3687 and miR-4417 to distinguish CRPC from primary PCa was further evaluated by ROC curve analysis (Fig. 1e, f). MiR-3195 (AUC 0.972), miR-3687 (AUC 0.942) and miR-4417 (AUC 0.908) possessed similar discriminatory power, whilst a combination of miR-3195 and miR-3687 showed improved accuracy (AUC 0.986). When the downregulated miR-205 was included in the analysis, the combination of miR-205, miR-3195 and miR-4417 had the highest AUC (0.994).

### Influence of androgens on the expression of CRPC-associated miRNAs

**Fig. 2:**
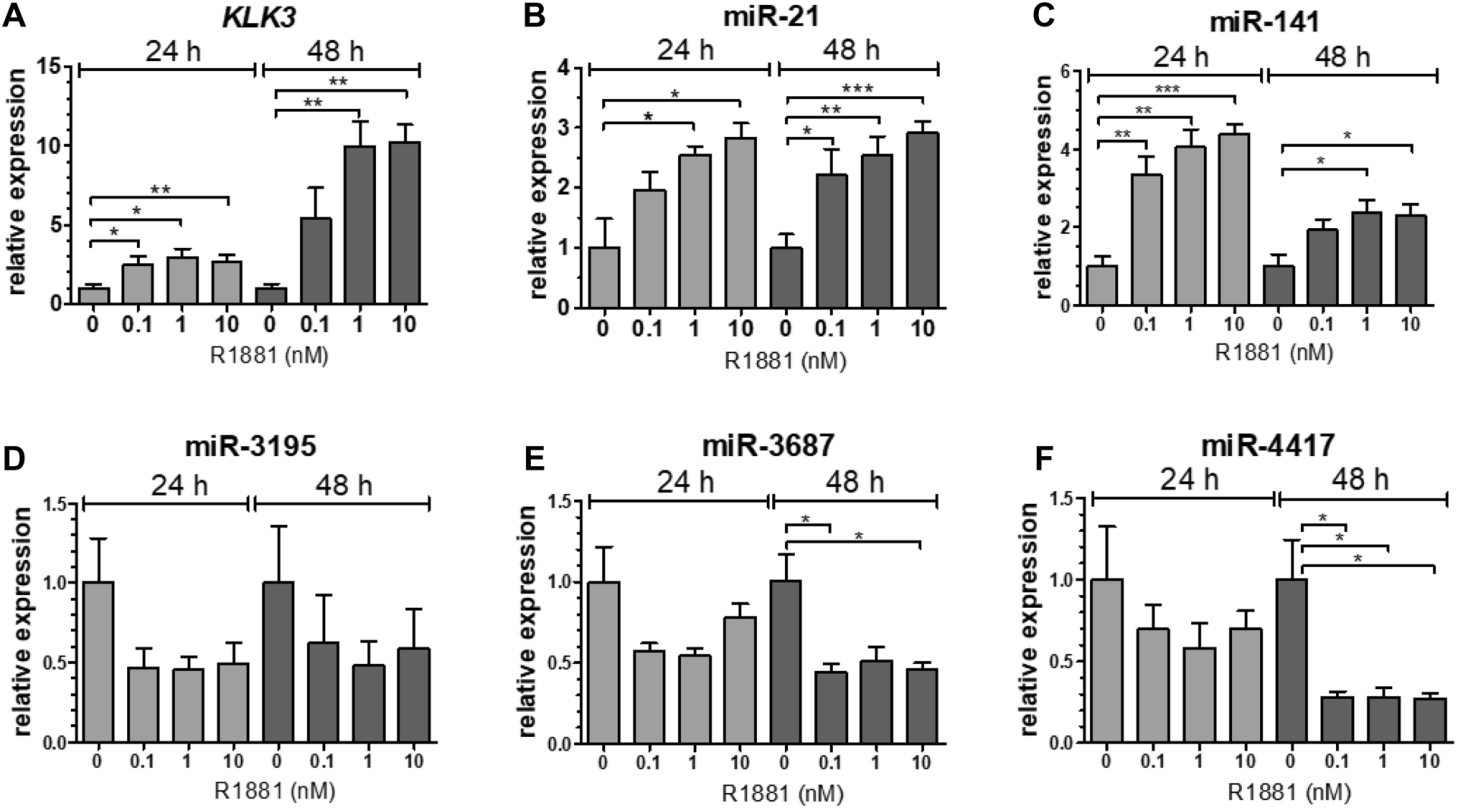
Androgen responsiveness of CRPC-associated miRNAs. Analysis of *KLK3* **a**, miR-21 **b**, miR-141 **c**, miR-3195 **d**, miR-3687 **e** and miR-4417 **f** expression in VCaP cells. VCaP cells were cultured for 3 days in androgen-free (CSS) medium, and then incubated in the presence of 0.1, 1 or 10 nM of synthetic androgen R1881. Total RNA was extracted 24 and 48 h after androgen stimulation. *KLK3* and miRNA levels were determined by reverse transcriptase real-time PCR analysis. *HPRT1* and RNU6 levels were used for normalization purposes. All experiments were repeated at least three times; p-values were calculated with the unpaired *t*-test (*p < 0.05; **p < 0.01; ***p < 0.001)

We evaluated the influence of androgens on CRPC-associated miRNA expression in PCa and CRPC cell lines. *KLK3* mRNA and two known androgen-regulated miRNAs, miR-21 and miR-141, were dose-dependently and significantly upregulated in DuCaP, LNCaP (data not shown) and VCaP cells in the presence of the synthetic androgen R1881 compared to vehicle treated cells (Fig. 2a–c). The CRPC-associated miRNAs miR-3195, miR-3687 and miR-4417 were (slightly) downregulated in the presence of R1881 in DuCaP and VCaP, but not in LNCaP cells (Fig. 2d–f, and data not shown). In AR-negative PC3 cells, expression levels of none of the miRNAs nor of *KLK3* were affected by androgen treatment.

### Effect of CRPC-associated miRNAs on survival, cell migration and cell invasion.

**Fig. 3:**
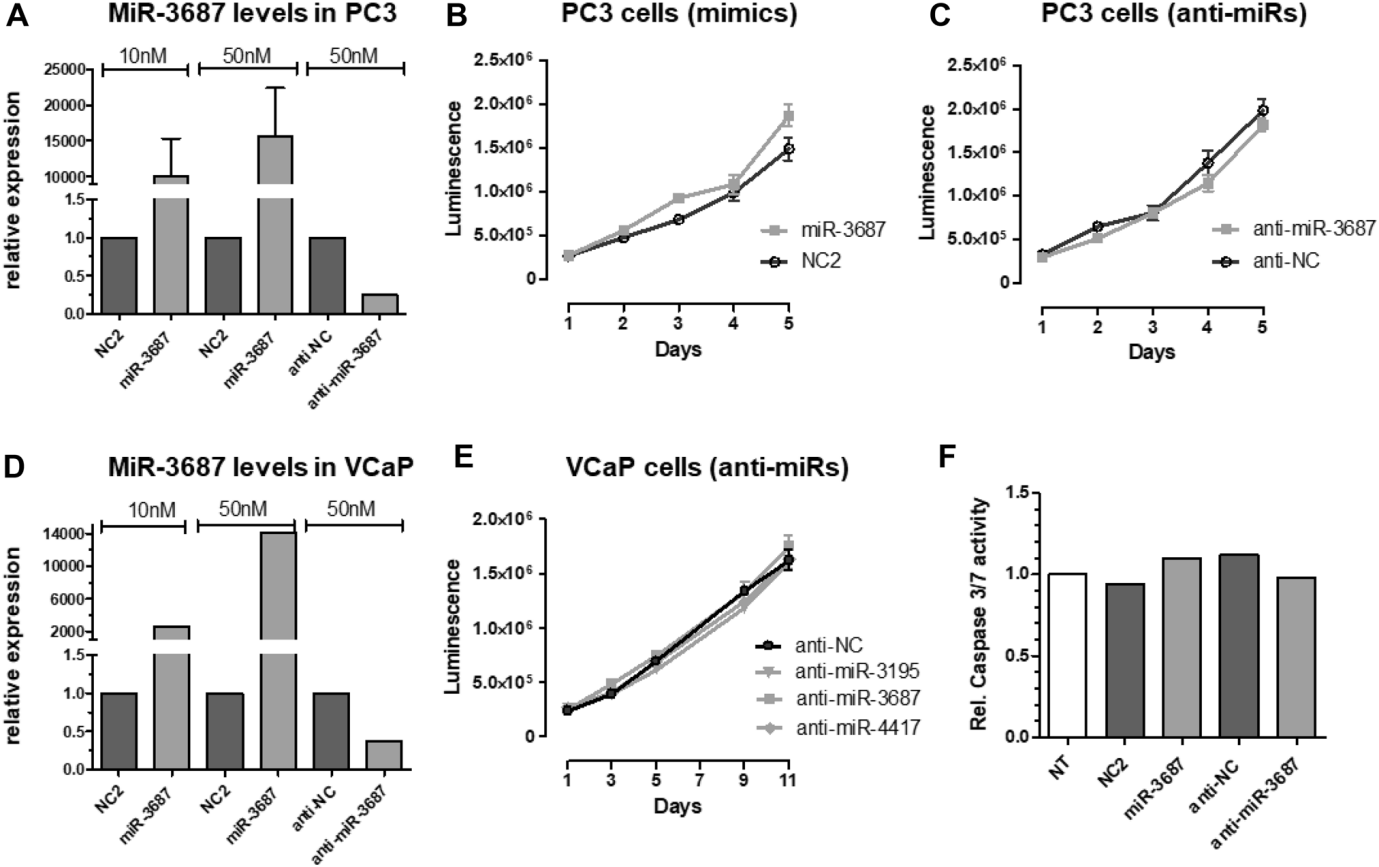
Effect of CRPC-associated miRNAs on cell survival. PC3 **a**–c, **f** and VCaP **d**–**e** cells were transfected with miRNA mimics (10–50 nM) or with anti-miR miRNA inhibitors (50 nM). Total RNA was extracted and the relative expression of miR-3687 was determined by stem-loop RT-qPCR analysis, using RNU6 for normalization **a**, **d**. Cell viability of miRNA mimic or anti-miR transfected cells was determined at different time points post-transfection **b**, **c**, **e**. Transfected cells were seeded 2 days post-transfection in 96-well plates. Cell viability was determined from day 1 until day 5 (PC3) or day 11 (VCaP), using CellTiter-Glo Assays. Apoptosis of miRNA mimic and anti-miR transfected PC3 cells was determined 2 days post transfection, using a Caspase-3/7 Assay **f**. *NT* non-transfected cells. Error bars indicate the standard deviation of at least 2 independent experiments

The endogenous expression of selected CRPC-associated miRNAs are high in LNCaP, VCaP and PC3 cells. Expression and function of the miRNAs were inhibited using anti-miR miRNA inhibitors. In addition, we overexpressed miR-3687, showing the lowest expression compared to miR-3195 and miR-4417, via transfection of synthetic miR-3687 precursors. MiRNA activation or inhibition was confirmed by stem-loop RT-qPCR analysis (Fig. 3a, d, and data not shown). MiRNA inhibition or overexpression did not affect cell survival (Fig. 3b, c and e), cell cycle distribution (data not shown), nor apoptosis-related Caspase-3/7 activation (Fig. 3f).

**Fig. 4:**
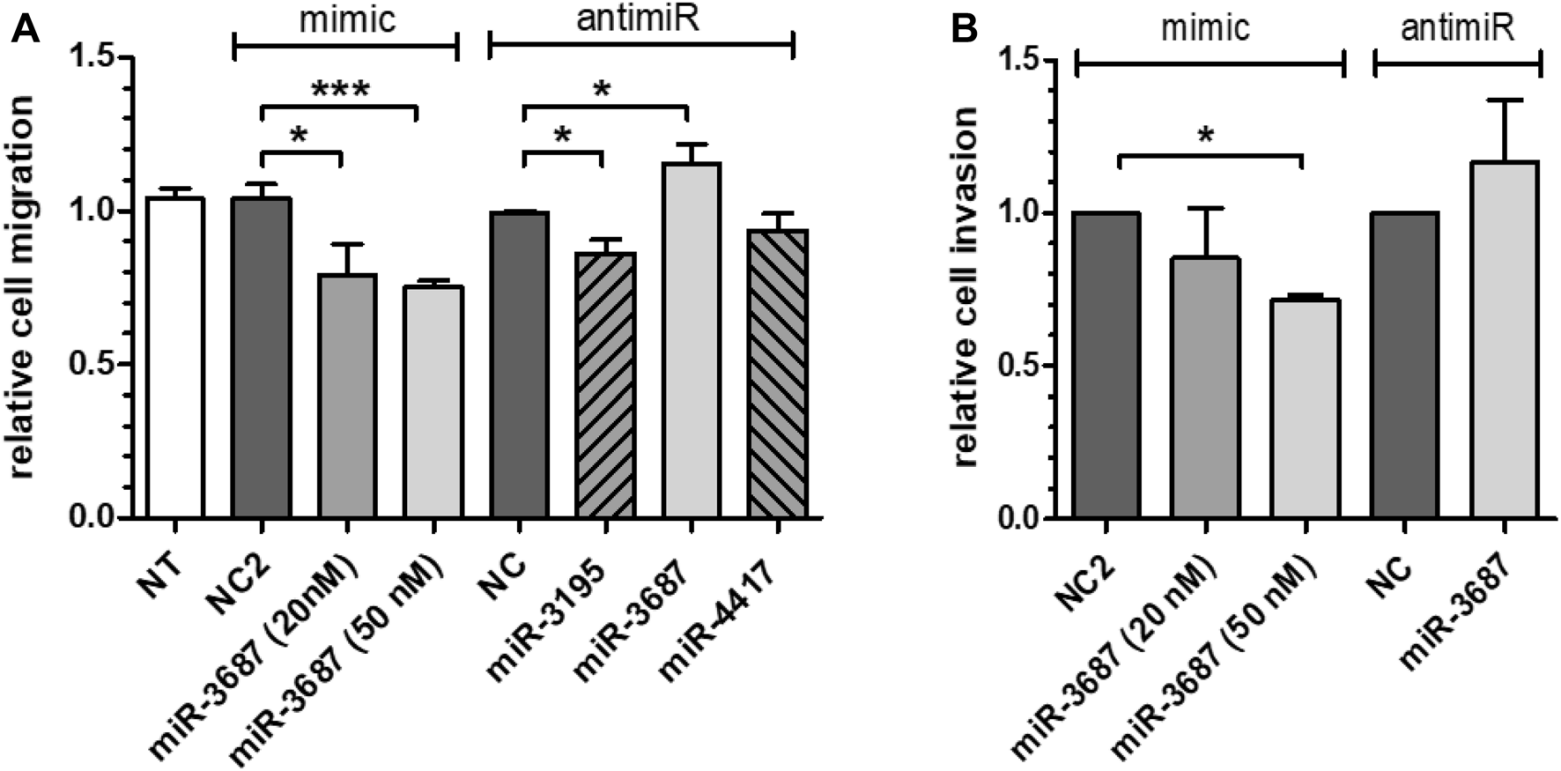
Effect of CRPC miRNAs on cell migration and cell invasion. Cell migration **a** was assessed by wound-healing “scratch” assays. Error bars indicate the standard deviation of at least four independent experiments performed in triplicate. Cell invasion **b** was determined using Matrigel-coated 8-µm cell invasion chambers. PC3 cells were transfected with miRNA mimics (10–50 nM) or with anti-miR miRNA inhibitors. Inhibition of cell invasion by a specific miRNA was calculated by dividing the percentage of invaded cells versus that of NC2 transfected cells. *NT* non-transfected cells. Error bars indicate the standard deviation of two independent experiments performed in duplicate; *p* values were calculated with the unpaired t-test (*p < 0.05; ***p < 0.001)

However, miR-3195 enhanced PC3 cell migration and anti-miR-3195 treatment reduced PC3 cell migration compared to negative control (NC)-treated cells (Fig. 4a). In contrast, miR-3687 significantly reduced cell migration and cell invasion of mimic transfected cells compared to NC control, which was confirmed by the enhanced cell migration and invasion found for anti-miR-3687 transfected cells (Fig. 4a, b). MiR-4417 had no significant effect on cell migration.

## Discussion

The molecular mechanisms involved in the progression of PCa to CRPC are still not fully understood. The identification of powerful gene regulators, such as miRNAs, will increase our understanding of the molecular mechanisms that underlie CRPC. Those miRNAs or their target genes may become useful agents for therapy. We have identified novel, so far unreported, miRNAs that are upregulated in CRPC. In particular miR-3195, miR-3687 and miR-4417 are able to discriminate CRPC from primary PCa with high accuracy. MiR-3195 and miR-3687 are also highly expressed in stroma, and whether these miRNAs are involved in the interaction between tumor epithelium and stroma remains to be investigated.

While miR-3195, miR-3687 and miR-4417 were significantly upregulated in CRPC specimens compared to primary PCa tissue in stem-loop RT-qPCR analysis, these miRNAs were downregulated in the microarray analysis. This discrepancy may be due to the fact that independent sample cohorts were used for both analyses. Mestdagh et al*.* showed that hybridization-based platforms (*e.g.* Affymetrix microarray) showed lower sensitivity compared to other platforms, and that qPCR technology is superior with respect to sensitivity and accuracy [17]. The differences in miRNA expression are not only dependent on the technology used for their detection, but also on the sequence of the transcript [18]. It has been described that miRNA levels that are measured by either the Agilent and Illumina quantitative RNA sequencing platforms are in strong agreement, but not with microarray analysis, the latter suffering from an over-representation of probes for guanine(G)-rich miRNAs. We recognize that, since our identified CRPC-associated miRNAs are G and C (cytosine)-rich, they and other GC-rich miRNAs may have cross-reacted. However, with the stem-loop RT-qPCR analysis they could be specifically and reliably quantified. Either the size of the cohorts used or the differences in miRNA detection technology, future studies may elucidate the cause of the discrepancies observed by expanding the number of samples in the cohorts and performing both stem-loop RT-qPCR and microarray analysis in the same sample set, if possible.

We also identified miR-205 to be the most downregulated miRNA in CRPC tissue, confirming previous results [14, 19]. In fact, downregulation of miR-205 in advanced PCa is the most consistent finding to date, across different studies. The functional role of the novel identified miRNAs is unknown. The AR signaling pathway is a relevant target in patients with metastatic PCa [20, 21]. Novel androgen-blocking targets such as enzalutamide and abiraterone acetate are new treatment options that have been demonstrated to improve survival of CRPC patients [22]. In this study, the CRPC-associated miRNAs were highly expressed in the cell line models, and androgens significantly downregulated the miRNAs in the CRPC-derived cell lines DuCaP and VCaP, but not in the PCa cell line LNCaP (Fig. 2). An explanation for this apparent paradoxical phenomenon remains unclear. Noteworthy is the fact that in this study the deregulated miRNAs were discovered in primary CRPC tissue and, while extensively characterized and validated as PCa models, the cell lines used are derived from metastatic tumors. It has been described that the AR and AR splice variants (AR-V) are relevant in CRPC development and progression [23], and that wild-type AR and AR-Vs both activate different, yet overlapping, sets of target genes [24–26]. The expression of the CRPC-associated miRNAs could be specifically activated by AR-Vs (in a low androgen environment), and ligand-activated wild-type AR may compete with AR-V-mediated miRNA activation. In LNCaP cells, AR-V levels are relatively low, and hence no such androgen and AR effect on miRNA expression was observed.

CRPC-associated miRNAs had no significant effect on proliferation and apoptosis of PC3 and VCaP cells. However, miR-3195 enhanced, while miR-3687 reduced PC3 cell migration and cell invasion. So far, there is not much known about the influence of miRNAs on cell migration and cell invasion in CRPC. Recently, it has been described that miR-663 is upregulated in CRPC tissue and that its overexpression promotes cell proliferation and invasion [27]. Overexpression of miR-146a, which is downregulated in CRPC tissue, inhibits cell growth, colony formation and cell migration [28]. The direct and indirect target genes of the CRPC-associated miRNAs remain unknown and further investigations are necessary to identify specific and relevant target mRNAs of miR-3195, miR-3687 and miR-4417 to prove their functional role in CRPC.

The identification of CRPC-specific miRNAs and their targets could lead to novel biomarkers for monitoring of CRPC patients, and for the prediction of treatment response. Further investigations are necessary to evaluate whether these novels identified CRPC-associated miRNAs are measurable in blood, serum and/or plasma. In a recent prospective clinical study, we have found that miR-3687 can be detected in plasma and whole blood, and that miR-3687 is a novel prognostic marker for enzalutamide treatment response in mCRPC patients [29]. With a personalized medicine strategy in mind, the role of these miRNA as prognostic markers for selecting anticancer treatments that meet individual patient needs should be further explored and validated.

In summary, we have identified novel upregulated miRNAs in CRPC tissue, two of which are involved in cell migration and cell invasion. Our data support the hypothesized involvement of miRNAs in PCa tumorigenesis and progression to CRPC. Further studies are needed to confirm the functional role of these novel ncRNAs in CRPC and to evaluate their applicability as biomarkers for CRPC.

## Supplementary Information

Below is the link to the electronic supplementary material.

Supplementary file1 (DOCX 767 kb)

## Data Availability

The data files supporting the findings of this study are available on request from the corresponding author.
