# Supplementary material for: Upregulation of miR-3195, miR-3687 and miR-4417 is associated with castration-resistant prostate cancer
Source: World J Urol. 2021 May 14;39(10):3789–97. doi: 10.1007/s00345-021-03723-4 (PMC8519832; doi:10.1007/s00345-021-03723-4)
Supplement: Supplementary file 1 — Supplementary file1 (DOCX 767 kb) [file 345_2021_3723_MOESM1_ESM.docx]

**SUPPLEMENTARY FIGURE AND TABLES**

**Title:**

Upregulation of miR-3195, miR-3687 and miR-4417 is associated with castration-resistant prostate cancer

**Journal:**

World Journal of Urology

**Authors:**

Rönnau C.G.H.^1,2^, Fussek S.^1,2^, Smit F.P.^3^, Aalders T.W.^1,4^, van Hooij O.^1,4^, Pinto P.M.C.^2^, Burchardt M.^2^, Schalken J.A.^1,4^ and Verhaegh G.W.^1,4,*^

^1^Dept. of Urology, Radboud university medical center, Nijmegen, The Netherlands

^2^Dept. of Urology, University medicine, Greifswald, Germany

^3^MDxHealth BV, Nijmegen, The Netherlands

^4^Radboud Institute for Molecular Life Sciences, Nijmegen, The Netherlands

**Corresponding author:**

Gerald W. Verhaegh PhD, Urological Research Laboratory, Department of Urology, Radboud university medical center, PO Box 9101, 6500 HB Nijmegen, the Netherlands. Tel.: +31-24-3610510; E-mail: gerald.verhaegh@radboudumc.nl

**Supplementary Figure 1: Hierarchical clustering of miRNA expression levels.**

(**A**) Hierarchical clustering is shown for mature miRNAs that were found to be differentially expressed between CRPC (red group) and primary PCa (orange group). Hierarchical clustering is based on miRNA expression as determined by Affymetrix GeneChip® miRNA array analysis. Red and green indicate low and high expression levels, resp. Red asterisks indicate the miRNAs that were validated by RT-qPCR.

**Supplementary Figure 2: Expression of candidate normalization miRNAs in human prostate tissue.**

Expression of miR-26a (**A, B**), miR-107 (**C, D**), miR-151-5p (**E, F**) and RNU6 (**H**) in human prostate tissues. Analysis was performed by Affymetrix GeneChip^®^ miRNA Array analysis (**A, C, E**) or by stem-loop RT-qPCR (**B, D, F, H**). For A, C and E, hybridization signals (2log values), and for B, D, F and H, Crossing point (Cp) values are shown. Average Cp and coefficient of variation (CV) values are displayed above each graph. (**G**) Table showing the NormFinder results for each miRNA separately, and the best miRNA combination. BPH, benign prostate hyperplasia; LG, low grade prostate cancer; HG, high grade prostate cancer; CRPC, castration-resistant prostate cancer.

**Supplementary Figure 3: Validation of normalization miRNAs in human prostate (cancer) tissue.**

Expression of RNU6 (**A),** miR-26a (**B**) and miR-107 (**C**) in human prostate tissues of the validation cohort (n=83 samples), used for the normalization of miRNA expression in tissue samples. Analysis were performed by stem-loop RT-qPCR. Cp-values are shown. (**D**) Table showing the NormFinder results for each miRNA separately, and the best miRNA combination. BPH, benign prostate hyperplasia; LG, low grade prostate cancer; HG, high grade prostate cancer; CRPC, castration-resistant prostate cancer.

**Supplementary Figure 4: Expression of miR-92b, miR-194, miR-451, miR-3156-5p and SNORD78 in human prostate (cancer) tissue.**

NcRNA expression analyses were performed by stem-loop RT-qPCR analysis. The results were normalized to the weighted average of miR-26a, miR-107 and RNU6 levels. MiR-92b (**A**), miR-194 (**B**), miR-205 (**C**), miR-451 (**D**), miR-3156-5p (**E**), miR-3195 (**F**), miR-3687 (**G**), miR-4417 (**H**) and SNORD78 (**I**) are significantly upregulated in CRPC tissue compared to primary PCa tissue. BPH, benign prostate hyperplasia; LG, low grade prostate cancer; HG, high grade prostate cancer; CRPC, castration-resistant prostate cancer.


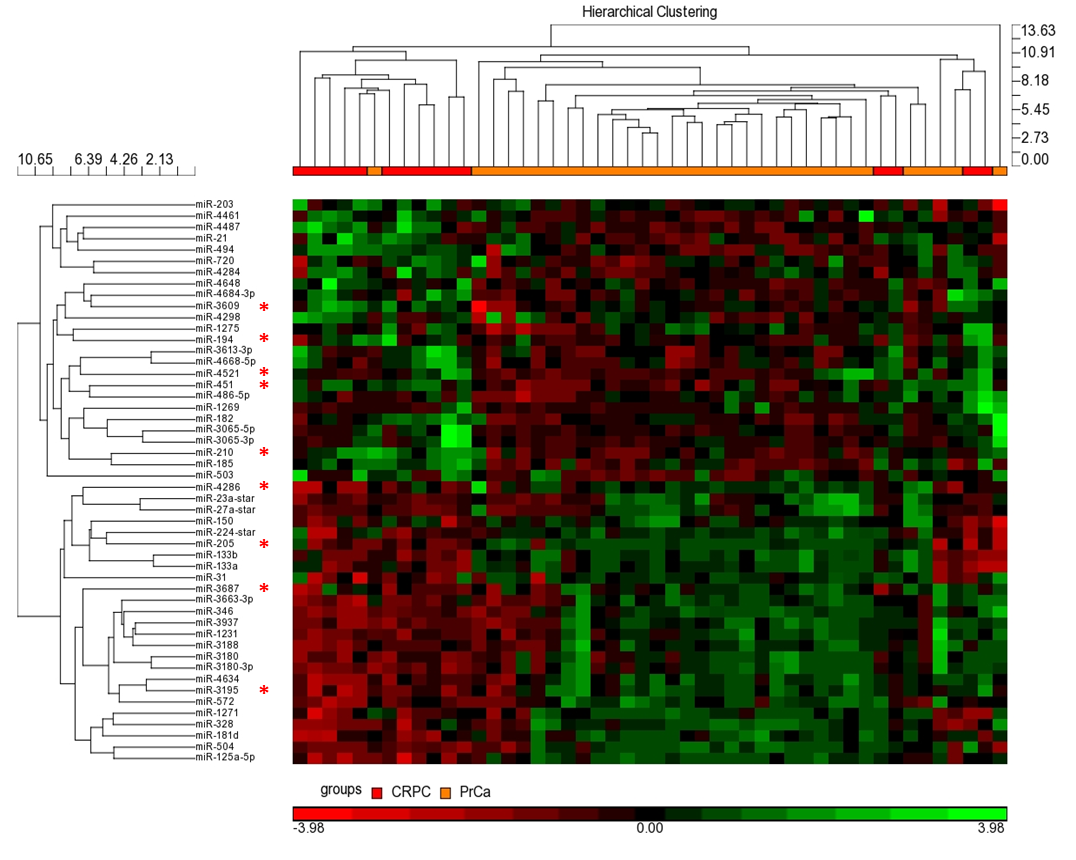
**Supplementary Figure 1**

**
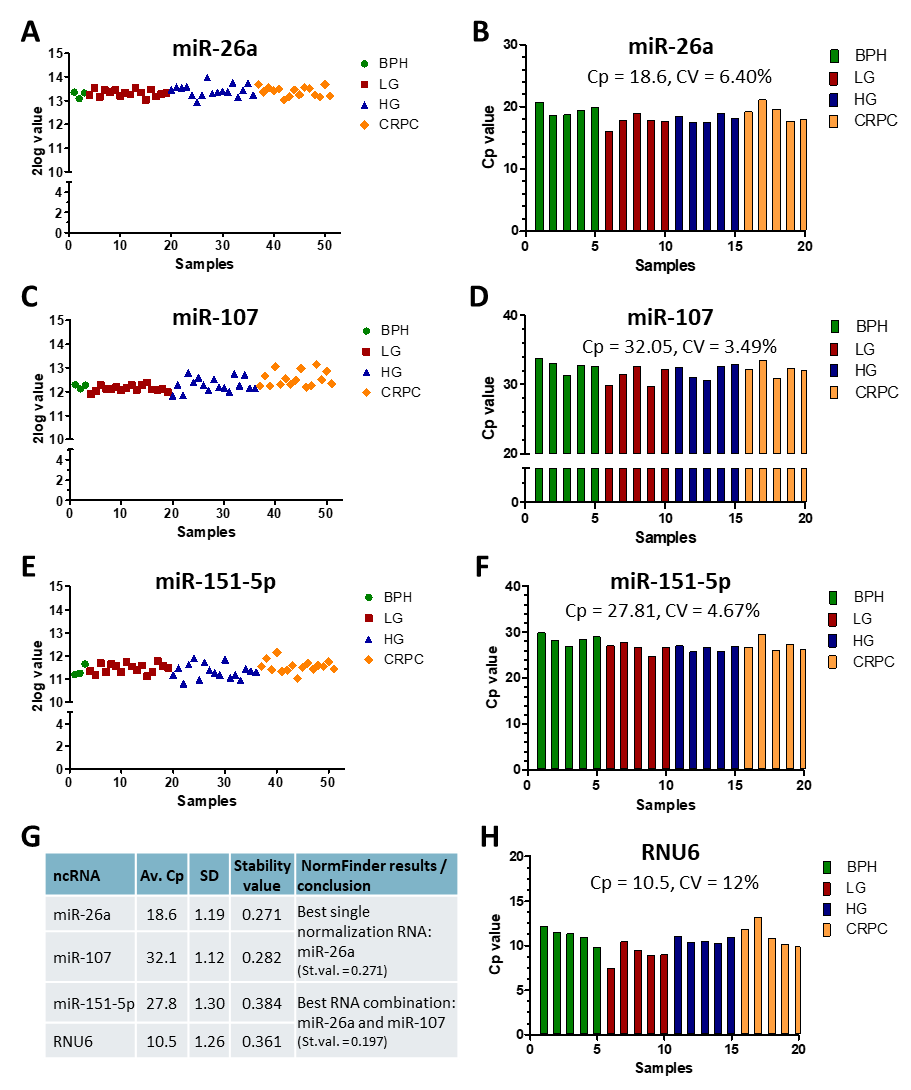
Supplementary Figure 2**


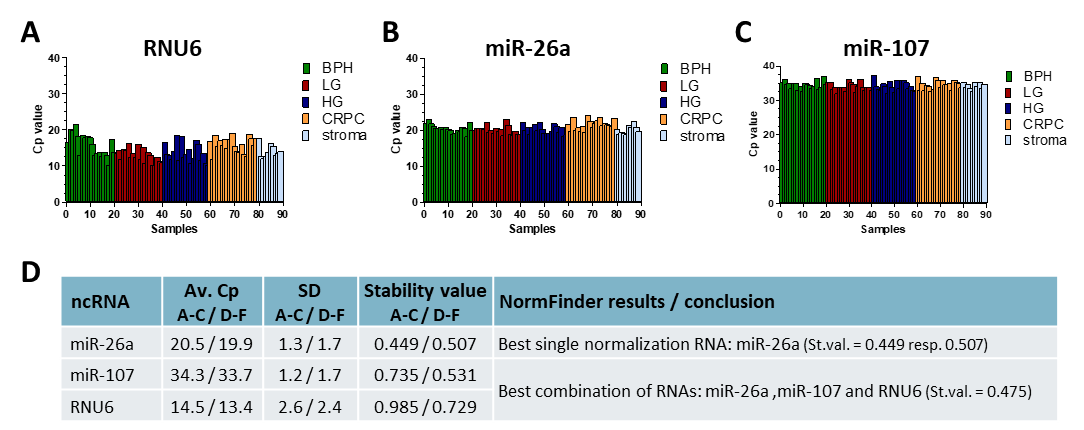
**Supplementary Figure 3**


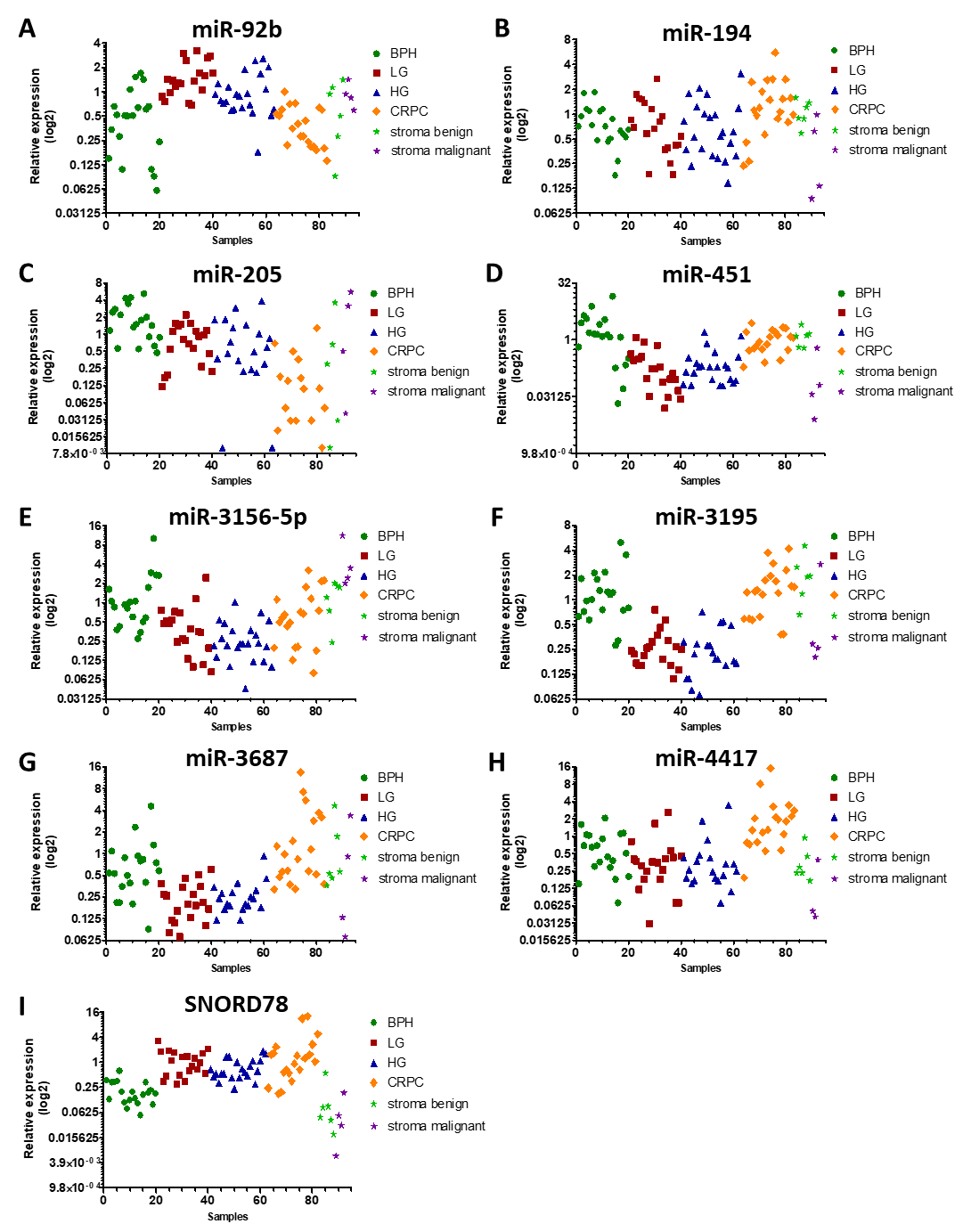
**Supplementary Figure 4**

**Supplementary Table 1:** Primers used for qPCR analysis.

| **(stem-loop) RT primers** | **Sequence (5’ to 3’)** | |
| --- | --- | --- |
| miR-21 | GTCGTATCCAGTGCAGGGTCCGAGGTATTCGCACTGGATACGACTCAACA | |
| miR-26a | GTCGTATCCAGTGCAGGGTCCGAGGTATTCGCACTGGATACGACAGCCTA | |
| miR-92b | CTCGTATCCAGTGCAGGGTCCGAGGTATTCGCACTGGATACGAGGGAGGC | |
| miR-107 | GTCGTATCCAGTGCAGGGTCCGAGGTATTCGCACTGGATACGACTGATAG | |
| miR-141 | GTCGTATCCAGTGCAGGGTCCGAGGTATTCGCACTGGATACGACCCATCT | |
| miR-151 5p | GTCGTATCCAGTGCAGGGTCCGAGGTATTCGCACTGGATACGACACTAGA | |
| miR-183 | GTCGTATCCAGTGCAGGGTCCGAGGTATTCGCACTGGATACGACAGTGAA | |
| miR-194 | GTCGTATCCAGTGCAGGGTCCGAGGTATTCGCACTGGATACGACTCCACA | |
| miR-205 | GTCGTATCCAGTGCAGGGTCCGAGGTATTCGCACTGGATACGACCAGACT | |
| miR-210 | GTCGTATCCAGTGCAGGGTCCGAGGTATTCGCACTGGATACGACTCAGCC | |
| miR-451 | GTCGTATCCAGTGCAGGGTCCGAGGTATTCGCACTGGATACGACAACTCA | |
| miR-708 | GTCGTATCCAGTGCAGGGTCCGAGGTATTCGCACTGGATACGACCCCAGC | |
| miR-3156-5p | GTCGTATCCAGTGCAGGGTCCGAGGTATTCGCACTGGATACGACTGTCTC | |
| miR-3195 | GTCGTATCCAGTGCAGGGTCCGAGGTATTCGCACTGGATACGACAACCCG | |
| miR-3609 | GTCGTATCCAGTGCAGGGTCCGAGGTATTCGCACTGGATACGACCAGCCA | |
| miR-3651 | GTCGTATCCAGTGCAGGGTCCGAGGTATTCGCACTGGATACGACTCATGT | |
| miR-3687 | CTCGTATCCAGTGCAGGGTCCGAGGTATTCGCACTGGATACGAGACGTCG | |
| miR-4286 | GTCGTATCCAGTGCAGGGTCCGAGGTATTCGCACTGGATACGACGGTACC | |
| miR-4417 | GTCGTATCCAGTGCAGGGTCCGAGGTATTCGCACTGGATACGACCCCTCC | |
| miR-4521 | GTCGTATCCAGTGCAGGGTCCGAGGTATTCGCACTGGATACGACCTGAGC | |
| sno-miR-78 | GTCGTATCCAGTGCAGGGTCCGAGGTATTCGCACTGGATACGACTCAGTG | |
| RNU6 | GTCATCCTTGCGCAGG | |
| SNORA84 | CCATGCATAAATCACAGGATTG | |
| SNORD49A | GTAGTCTTCGTCAGTTATCGC | |
| SNORD78 | GTGTTACCTTTGTCTACATGC | |
| **miRNA qPCR primers** | **Forward (5’ to 3’)** | **Reverse (5’ to 3’)** |
| miR-21 | GCCCGCTAGCTTATCAGACTGATG | GTGCAGGGTCCGAGGT |
| miR-26a | GCCCGCTTCAAGTAATCCAGGAT |  |
| miR-92b | TGCCAGTATTGCACTCGTCCCG |  |
| miR-107 | TGCCAGAGCAGCATTGTACAGG |  |
| miR-141 | GCCCGCTAACACTGTCTGGTAAAG |  |
| miR-151-5p | TGCCAGTCGAGGAGCTCACAGTC |  |
| miR-183 | TGCCAGTATGGCACTGGTAGAA |  |
| miR-194 | TGCCAGTGTAACAGCAACTCCA |  |
| miR-205 | TGCCAGTCCTTCATTCCACCGG |  |
| miR-210 | TGCAAGCTGTGCGTGTGACAGC |  |
|  |  |  |
| **miRNA qPCR primers** | **Forward (5’ to 3’)** | **Reverse (5’ to 3’)** |
| miR-451 | GCCCGCAAACCGTTACCATTACT | GTGCAGGGTCCGAGGT |
| miR-708 | GCCCGCAAGGAGCTTACAATCTAG |  |
| miR-3156-5p | TGCCAGAAAGATCTGGAAGTGG |  |
| miR-3195 | TGCAAGCGCGCCGGGCCCGGGT |  |
| miR-3609 | GCCCGCCAAAGTGATGAGTAATA |  |
| miR-3651 | TGCAAGCATAGCCCGGTCGCT |  |
| miR-3687 | TGCAAGCCCGGACAGGCGTTCGT |  |
| miR-4286 | GCCCGCACCCCACTCCTGGUAC |  |
| miR-4417 | TGCAAGGGTGGGCTTCCCGGAG |  |
| miR-4521 | TGCCAGGCTAAGGAAGTCCTGTG |  |
| sno-miR-78 | GCCGCAATGAGCATGTAGACAAAGG |  |
| **Gene qPCR primers** | **Forward (5’ to 3’)** | **Reverse (5’ to 3’)** |
| *KLK3* | CAGCTGCCCACTGCATCAGG | GGGTGAACTTGCGCACACAC |
| *HPRT1* | CTCAACTTTAACTGGAAAGAATGTC | TCCTTTTCACCAGCAAGCT |
| RNU6 | CGCTTCGGCAGCACATATAC | AGGGGCCATGCTAATCTTCT |
| SNORA84 | GGTTGCTGGATGCTGTTGT | CCATCGAATCCACTGGAGAG |
| SNORD49A | ATCACTAATAGGAAGTGCCGTC | TAGTCTTCGTCAGTTATCGCTTCT |
| SNORD78 | TGTTGATCAAATGTCTGACCTG | TGTTACCTTTGTCTACATGCTCATT |

List of primers and oligonucleotides used in this study. Underlined sequences, miR-complementary nucleotides in stem-loop RT primers. All sequences 5’ to 3’ direction.

**Supplementary Table 2:** miRNA mimics and anti-miR miRNA inhibitors used in this study.

| **miRNA precursors (‘mimics’)** | **Ambion catalog number** | **Mature miR sequence (5’ to 3’)** |
| --- | --- | --- |
| negative control #2 (NC2) | AM17111 |  |
| miR-3687 | AM17100 (ID: PM20273) | CCCGGACAGGCGUUCGUGCGACGU |
| **Anti-miR miRNA inhibitors** | **Ambion catalog number** | **Mature miR sequence (5’ to 3’)** |
| Negative control #1 (Anti-NC) | AM17010 |  |
| Anti-miR-3195 | AM17000 (ID: AM18897) | CGCGCCGGGCCCGGGUU |
| Anti-miR-3687 | AM17000 (ID: AM20273) | CCCGGACAGGCGUUCGUGCGACGU |
| Anti-miR-4417 | AM17000 (ID: AM20921) | GGUGGGCUUCCCGGAGGG |

**Supplementary Table 3:** Clinical characteristics of primary prostate cancer patients included in this study.

|  | **Profiling** | | **Validation** | |
| --- | --- | --- | --- | --- |
| **Tumor classification** | LG | HG | LG | HG |
|  | (n=16) | (n=17) | (n=20) | (n=23) |
| **Gleason Score** |  |  |  |  |
| 4 | 1 | 0 | 0 | 0 |
| 5 | 2 | 0 | 0 | 0 |
| 6 | 13 | 0 | 20 | 0 |
| 7 | 0 | 2 | 0 | 6 |
| 8 | 0 | 8 | 0 | 9 |
| 9 | 0 | 1 | 0 | 6 |
| 10 | 0 | 6 | 0 | 1 |
| **Clinical T stage** |  |  |  |  |
| T1 | 0 | 0 | 0 | 0 |
| T2 | 8 | 2 | 6 | 6 |
| T3 | 5 | 12 | 4 | 7 |
| T4 | 1 | 3 | 2 | 1 |
| Tx | 2 | 0 | 8 | 8 |
| **Clinical N stage** |  |  |  |  |
| N0 | 12 | 14 | 9 | 11 |
| N1 | 1 | 0 | 0 | 0 |
| Nx | 3 | 3 | 11 | 12 |
| **Clinical M stage** |  |  |  |  |
| M0 | 14 | 15 | 12 | 14 |
| M1 | 0 | 2 | 0 | 0 |
| Mx | 2 | 0 | 8 | 9 |
| **Follow up (2-17 years):** |  |  |  |  |
| Prostatectomy as curative treatment | 6 | 1 | 5 | 6 |
| cured after adjuvant radiotherapy | 4 | 0 | 2 | 1 |
| biochemical recurrence | 3 | 10 | 3 | 7 |
| metastatic prostate cancer in follow up | 1 | 9 | 2 | 5 |
| lost to follow up (< 2 years) | 3 | 4 | 9 | 9 |

TNM (Tumor Node Metastasis) classification of prostate cancer: T1, clinical unapparent tumor; T2, Tumor confined within in the prostate; T3, Tumor extends through the prostate capsule or invades the seminal vesicles; T4, Tumor is fixed or invades adjacent structures other than seminal vesicles; Tx, primary tumor cannot be assessed; N0, no regional lymph node metastasis; N1, regional lymph node metastasis; Nx, regional lymph nodes cannot be assessed; M0, no distant metastasis; M1, distant metastasis; Mx, distant metastasis cannot be assessed. HG, high grade prostate cancer; LG, low grade prostate cancer.

**Supplementary Table 4:** Selection of differentially expressed miRNAs in prostate cancer specimens identified by Affymetrix GeneChip miRNA Array analysis for validation.

| **Profile** | **Description** | **miRNA** | **Fold change** | **p-value** |
| --- | --- | --- | --- | --- |
|  |  | miR-451 | 3.48 | 0.0004 |
|  | **upregulated in** | miR-210 | 3.25 | < 0.0001 |
|  | **CRPC vs. primary** | miR-4521 | 2.70 | 0.0030 |
|  | **prostate cancer** | miR-3609 | 2.55 | < 0.0001 |
|  |  | miR-194 | 1.62 | 0.0119 |
| **Profile 1** |  | miR-3156-5p | 1.50 | 0.0425 |
|  |  | miR-205 | 8.94 | 0.0003 |
|  | **downregulated in** | miR-3687 | 4.82 | 0.0004 |
|  | **CRPC vs. primary** | miR-4286 | 3.64 | 0.0001 |
|  | **prostate cancer** | miR-3195 | 3.09 | < 0.0001 |
|  |  | miR-92b | 1.96 | 0.0045 |
|  |  | miR-183 | 32.9 | < 0.0001 |
|  |  | miR-4417 | 29.6 | < 0.0001 |
|  | **upregulated in** | miR-3687 | 19.3 | 0.0003 |
| **Profile 2** | **primary prostate** | miR-375 | 14.5 | < 0.0001 |
|  | **vs. BPH** | miR-92b | 14.3 | < 0.0001 |
|  |  | miR-4286 | 12.9 | < 0.0001 |
|  |  | miR-4443 | 12.6 | < 0.0001 |
|  |  | miR-182 | 12.3 | < 0.0001 |
|  |  | miR-3195 | 12.0 | < 0.0001 |
|  |  | miR-708 | 11.2 | < 0.0001 |
|  |  | miR-3651 | 8.7 | < 0.0001 |
|  |  | miR-194 | 6.8 | < 0.0001 |

BPH, benign prostate hyperplasia; CRPC, castration-resistant prostate cancer; P-values were calculated based on the unpaired two-tailed t-test.

**Supplementary Table 5:** Differentially expressed miRNAs 1.5-fold down and upregulated in CRPC vs. primary prostate cancer specimens identified by Affymetrix GeneChip miRNA Array analysis.

| **CRPC vs. Primary PCa (DOWN)** | | |  | **CRPC vs. Primary PCa (UP)** | | |  |  |
| --- | --- | --- | --- | --- | --- | --- | --- | --- |
| **miRNA** | **Fold-change** | **p-value** | **#** | **miRNA** | **Fold-change** | **p-value** |  |  |
| miR-205 | -9.94 | 0.000258 | 1 | miR-4668-5p | 4.19 | 0.000018 |  |  |
| miR-3687 | -4.82 | 0.000375 | 2 | miR-451 | 3.48 | 0.000363 |  |  |
| miR-133b | -4.59 | 0.000001 | 3 | miR-210 | 3.25 | 0.000063 |  |  |
| miR-133a | -4.02 | 0.000292 | 4 | miR-3613-3p | 3.05 | 0.000004 |  |  |
| miR-4286 | -3.64 | 0.000133 | 5 | miR-4521 | 2.70 | 0.002986 |  |  |
| miR-27a-star | -3.48 | 0.000140 | 6 | miR-3609 | 2.55 | 0.000030 |  |  |
| miR-125a-5p | -3.22 | 0.000001 | 7 | miR-4487 | 2.23 | 0.000002 |  |  |
| miR-3188 | -3.11 | 0.000357 | 8 | miR-494 | 1.96 | 0.001490 |  |  |
| miR-3195 | -3.09 | 0.000043 | 9 | miR-486-5p | 1.81 | 0.095685 |  |  |
| miR-504 | -2.76 | 0.000717 | 10 | miR-4284 | 1.79 | 0.053663 |  |  |
| miR-4634 | -2.74 | 0.000060 | 11 | miR-503 | 1.78 | 0.051199 |  |  |
| miR-31 | -2.71 | 0.013863 | 12 | miR-3065-3p | 1.76 | 0.023166 |  |  |
| miR-572 | -2.66 | 0.000460 | 13 | miR-21 | 1.75 | 0.001230 |  |  |
| miR-1271 | -2.55 | 0.000019 | 14 | miR-1269 | 1.74 | 0.100020 |  |  |
| miR-3180-3p | -2.52 | 0.000144 | 15 | miR-1275 | 1.68 | 0.005606 |  |  |
| miR-1231 | -2.49 | 0.003766 | 16 | miR-203 | 1.66 | 0.102700 |  |  |
| miR-224-star | -2.46 | 0.000316 | 17 | miR-4461 | 1.66 | 0.023888 |  |  |
| miR-328 | -2.39 | 0.000311 | 18 | miR-194 | 1.62 | 0.011866 |  |  |
| miR-346 | -2.39 | 0.000408 | 19 | miR-182 | 1.60 | 0.010826 |  |  |
| miR-23a-star | -2.37 | 0.000078 | 20 | miR-3065-5p | 1.58 | 0.058142 |  |  |
| miR-3663-3p | -2.32 | 0.001085 | 21 | miR-185 | 1.54 | 0.000047 |  |  |
| miR-181d | -2.28 | 0.000009 | 22 | miR-720 | 1.54 | 0.191756 |  |  |
| miR-3937 | -2.27 | 0.001078 | 23 | miR-4648 | 1.53 | 0.000534 |  |  |
| miR-3180 | -2.27 | 0.000912 | 24 | miR-4684-3p | 1.51 | 0.001629 |  |  |
| miR-150 | -2.26 | 0.002291 | 25 | miR-4298 | 1.50 | 0.023506 |  |  |
| miR-197 | -2.21 | 0.001083 |  | miR-3156-5p | 1.50 | 0.042460 |  |  |
| miR-4324 | -2.19 | 0.000599 |  |  |  |  |  |  |
| miR-29c-star | -2.17 | 0.006191 |  |  |  |  |  |  |
| miR-1225-5p | -2.16 | 0.000872 |  |  |  |  |  |  |
| miR-200b-star | -2.16 | 0.039390 |  | **CRPC vs. Primary PCa (DOWN)** | | |  |  |
| miR-148a-star | -2.15 | 0.000406 |  | **miRNA** | **Fold-change** | **p-value** |  |  |
| miR-1180 | -2.15 | 0.010700 |  | *continued from left column(s)* | | |  |  |
| miR-4532 | -2.14 | 0.000033 |  | miR-363-star | -1.72 | 6.732 E-03 |  |  |
| miR-128 | -2.12 | 0.000116 |  | miR-222 | -1.72 | 1.556 E-03 |  |  |
| miR-4486 | -2.10 | 0.006184 |  | miR-191-star | -1.72 | 6.789 E-04 |  |  |
| miR-663b | -2.10 | 0.007677 |  | miR-4758-5p | -1.72 | 1.005 E-02 |  |  |
| miR-4750 | -2.09 | 0.000618 |  | miR-29b-1-star | -1.71 | 3.187 E-02 |  |  |
| miR-150-star | -2.09 | 0.000729 |  | miR-148b | -1.71 | 1.108 E-02 |  |  |
| miR-4783-3p | -2.07 | 0.000165 |  | miR-4690-5p | -1.70 | 1.225 E-02 |  |  |
| miR-212 | -2.07 | 0.000188 |  | miR-3124-5p | -1.69 | 1.003 E-03 |  |  |
| miR-3187-3p | -2.07 | 0.001904 |  | miR-574-3p | -1.69 | 2.910 E-03 |  |  |
| miR-4767 | -2.06 | 0.003919 |  | miR-3621 | -1.68 | 1.060 E-02 |  |  |
| miR-1909 | -2.05 | 0.000456 |  | miR-130a | -1.67 | 8.286 E-04 |  |  |
| miR-1273f | -2.05 | 0.002852 |  | miR-4253 | -1.67 | 3.144 E-03 |  |  |
| miR-1910 | -2.05 | 0.007563 |  | miR-214-star | -1.67 | 1.944 E-02 |  |  |
| miR-1909-star | -2.04 | 0.000212 |  | miR-223 | -1.66 | 1.654 E-02 |  |  |
| miR-4322 | -2.03 | 0.000485 |  | miR-1 | -1.65 | 9.226 E-02 |  |  |
| miR-183 | -2.02 | 0.022870 |  | miR-200a | -1.65 | 1.284 E-01 |  |  |
| miR-181c | -1.99 | 0.000362 |  | miR-138 | -1.64 | 4.737 E-02 |  |  |
| miR-1246 | -1.99 | 5.263 E-02 |  | miR-505 | -1.64 | 1.721 E-02 |  |  |
| miR-4665-5p | -1.98 | 1.043 E-03 |  | miR-602 | -1.63 | 1.596 E-02 |  |  |
| miR-4443 | -1.98 | 6.289 E-03 |  | miR-4707-5p | -1.62 | 4.709 E-02 |  |  |
| miR-4741 | -1.97 | 9.397 E-03 |  | miR-154 | -1.62 | 2.233 E-02 |  |  |
| miR-92b | -1.96 | 4.516 E-03 |  | miR-4721 | -1.61 | 1.065 E-02 |  |  |
| miR-4417 | -1.95 | 2.574 E-02 |  | miR-940 | -1.61 | 9.217 E-04 |  |  |
| miR-4436b-5p | -1.94 | 8.160 E-03 |  | miR-132 | -1.61 | 4.696 E-03 |  |  |
| miR-206 | -1.93 | 1.865 E-01 |  | miR-766 | -1.60 | 2.393 E-03 |  |  |
| miR-4800-3p | -1.91 | 1.000 E-03 |  | miR-378d | -1.60 | 1.806 E-02 |  |  |
| miR-3185 | -1.91 | 2.661 E-02 |  | miR-1280 | -1.59 | 7.418 E-02 |  |  |
| miR-371b-5p | -1.90 | 1.813 E-02 |  | miR-4484 | -1.57 | 2.684 E-02 |  |  |
| miR-125b-1-star | -1.90 | 2.638 E-03 |  | miR-1238 | -1.57 | 1.930 E-04 |  |  |
| miR-2277-3p | -1.88 | 1.894 E-03 |  | miR-4449 | -1.56 | 6.503 E-02 |  |  |
| miR-148a | -1.88 | 6.303 E-03 |  | miR-4725-3p | -1.56 | 5.775 E-03 |  |  |
| miR-628-3p | -1.85 | 3.095 E-03 |  | miR-125b | -1.55 | 7.487 E-04 |  |  |
| miR-4649-5p | -1.85 | 1.988 E-03 |  | miR-3135b | -1.55 | 5.559 E-02 |  |  |
| miR-665 | -1.83 | 3.773 E-03 |  | miR-99b-star | -1.55 | 4.136 E-02 |  |  |
| miR-99a-star | -1.81 | 7.263 E-03 |  | miR-422a | -1.55 | 3.323 E-02 |  |  |
| miR-939 | -1.80 | 2.026 E-03 |  | miR-484 | -1.55 | 3.164 E-02 |  |  |
| miR-1228 | -1.79 | 1.940 E-05 |  | miR-1307 | -1.54 | 5.881 E-02 |  |  |
| miR-1281 | -1.78 | 1.206 E-02 |  | miR-663 | -1.54 | 2.682 E-02 |  |  |
| miR-30c-2-star | -1.78 | 2.022 E-02 |  | miR-4695-5p | -1.53 | 3.163 E-02 |  |  |
| miR-4462 | -1.76 | 6.634 E-04 |  | miR-4674 | -1.52 | 7.479 E-02 |  |  |
| miR-532-3p | -1.76 | 6.992 E-03 |  | miR-4734 | -1.52 | 1.177 E-01 |  |  |
| miR-378-star | -1.74 | 1.274 E-02 |  | miR-1273d | -1.51 | 1.816 E-02 |  |  |
| miR-4269 | -1.73 | 1.217 E-02 |  | miR-5096 | -1.51 | 1.655 E-02 |  |  |
| miR-125b-2-star | -1.73 | 7.760 E-03 |  | miR-146a | -1.51 | 7.478 E-02 |  |  |
| miR-29b | -1.73 | 4.328 E-02 |  | miR-181a-2-star | -1.51 | 3.811 E-02 |  |  |
| miR-4745-5p | -1.72 | 1.568 E-02 |  | miR-378g | -1.51 | 3.095 E-02 |  |  |
| *continued in right column(s)* | | |  | miR-2110 | -1.50 | 1.034 E-01 |  |  |
|  | | |  |  |  |  |  |  |

CRPC, castration-resistant prostate cancer; +, upregulated ncRNAs in CRPC compared to primary prostate cancer tissue; -, downregulated ncRNAs in CRPC compared to primary prostate cancer; P-values were calculated based on the unpaired two-tailed t-test. Top-25 up and down regulated miRNAs, used for Hierarchical clustering (Suppl. Figure 1), are highlighted in green and red, resp.
